# Evaluation of dopamine transporter density in healthy Brazilians using Tc-99m TRODAT-1 SPECT

**DOI:** 10.1097/MD.0000000000027192

**Published:** 2021-10-01

**Authors:** Marilia Alves dos Reis, Ary Gadelha, André C. Felício, Marcelo Queiroz Hoexter, Ilza Rosa Batista, Pedro Braga-Neto, Mariana Calzavara, Daniel Alves Cavagnolli, Cinthia Higuchi, Melissa Furlaneto Lellis Leite, Solange Amorim Nogueira, Jairo Wagner, Mario Luiz Vieira Castiglioni, Ming Chi Shih, Rodrigo Affonseca Bressan

**Affiliations:** a Laboratory of Integrative Neuroscience, Universidade Federal de São Paulo (LiNC- EPM/UNIFESP), São Paulo, Brazil; b Department of Psychiatry, Universidade Federal de São Paulo, São Paulo, Brazil; c Department of Diagnostic Imaging, Universidade Federal de São Paulo (DDI/UNIFESP), São Paulo, Brazil; d Schizophrenia Program of the Department of Psychiatry, Universidade Federal de São Paulo (PROESQ – EPM/UNIFESP), São Paulo, Brazil; e Hospital Israelita Albert Einstein, São Paulo, Brazil; f Neurology Division, Department of Clinical Medicine, Universidade Federal do Ceará, Ceará, Brazil; g Center of Health Sciences, Universidade Estadual do Ceará, Ceará, Brazil; h Department of Psychobiology, Universidade Federal de São Paulo, São Paulo, Brazil.

**Keywords:** binding potential, dopamine transporter, healthy individuals, single photon emission computed tomography, technetium-99 metastable TRODAT-1

## Abstract

The presynaptic dopamine transporter (DAT) modulates the uptake of dopamine by regulating its concentration in the central nervous system. We aimed to evaluate the DAT binding potential (DAT-BP) in a sample of healthy Brazilians through technetium-99 metastable TRODAT-1 single-photon emission computed tomography imaging.

We selected 126 healthy individuals comprising 72 men and 54 women, aged 18 to 80 years. We conducted semi-quantitative evaluation in transaxial slices, following which we identified the regions of interest in the striatal region using the occipital lobe as a region of non-specific DAT-BP.

We found a decrease in DAT-BP in healthy individuals aged over 30 years, culminating in a 42% mean reduction after 80 years. There was no difference in the decrease by age group between the right (linear regression test [*R*^2^] linear = 0.466) and left striatum (*R*^2^ linear = 0.510). Women presented a higher DAT-BP than men (women: *R*^2^ linear = 0.431; men: *R*^2^ linear = 0.457); nonetheless, their decrease by age group was equal to that in men.

Our study sheds light on important DAT-BP findings in healthy Brazilian subjects. Our results will facilitate understanding of brain illnesses that involve the dopamine system, such as neuropsychiatric disorders.

## 1 Introduction

The presynaptic dopamine transporter (DAT) is involved in regulating synaptic dopamine levels by a reuptake mechanism.^[1,2]^ The striatum is a rich in dopamine. Thus, it has a high density of presynaptic DAT.^[3]^ Findings from post-mortem examinations report on a reduction in the density of DAT in the striatum of patients with Parkinson disease (PD) and Alzheimer disease. Thus, measurement of the decrease in DAT may be an indicator of the loss of dopaminergic neurons.^[4,5]^

Several ligands for positron emission tomography and single-photon emission computed tomography (SPECT) imaging have displayed a high binding affinity and excellent imaging characteristics for DAT.^[6]^ These imaging techniques require cyclotron produced radionuclides, such as carbon-11, fluor-18, and iodine-123, which limits their availability and use in routine clinical diagnosis.^[7]^ Furthermore, technetium-99 metastable (Tc-99m) radiopharmaceuticals are used for nuclear medicine procedures. Current diagnostic medical imaging instruments are optimized for the gamma emission of Tc-99m. Therefore, a Tc-99m tracer for in vivo binding with DAT would be ideal for a routine clinical study in humans.^[8,9]^

The TRODAT-1 is a tropane derivative labeled with Tc-99m (Tc-99m TRODAT-1). It crosses the blood brain barrier and has a high affinity for DAT.^[2,10,11]^ SPECT scintigraphy with Tc-99m TRODAT-1 can generate images of specific sites of DAT. Tc-99m TRODAT-1 shows similar binding and imaging efficiency at much lower costs compared with other tracers, thus being more advantageous.^[9]^

Tc-99m TRODAT-1 is being used in Brazil to investigate dopaminergic neurotransmission in PD.^[12–17]^ However, there is lack of literature on the evaluation of DAT in healthy Brazilians for better understanding of dopaminergic neurodegeneration. Thus, we aimed to evaluate DAT density in a sample of healthy Brazilians using the Tc-99m TRODAT-1 SPECT image analysis.

## 2 Methods

### 2.1 Sample

We selected images from the Tc-99m TRODAT-1 image database of the Laboratory of Integrative Neuroscience (LiNC-EPM/UNIFESP). This database contains images of normal volunteers acquired from 2006 to 2014. The inclusion criteria were as follows: no neurological disease, no severe intellectual disability, no comorbidities with Axis I disorders according to Diagnostic and Statistical Manual of Mental Disorders IV, and no artefacts in the images. Our study was approved by the Research Ethics Committee of the Universidade Federal de São Paulo (UNIFESP) (protocol number 0315/2017). The informed consent was obtained from each subject at the time of enrollment for imaging data.

### 2.2 Image acquisition

We acquired all images on a double-headed gamma camera equipped with ultra-high-resolution fan beam collimators (General Electric Healthcare—GE Discovery and GE Hawkeye Infinia). We began emission scans 4 hours after the intravenous injection of 814–888 MBq/2 mL Tc-99m TRODAT-1. The Institute of Nuclear Energy Research (Taiwan, China) produced the TRODAT-1 kits. Following their labeling according to a previously described methodology,^[18,19]^ we extensively validated them.^[14,16]^

The acquisitions were made with a matrix of 128 × 128 × 16 on a circular orbit with 128 steps and 3600 rotations, 30 seconds by projection, with a zoom factor of 1.45. We used a sinogram to control the quality of the exam, thus revealing possible subject movements during the acquisition.

### 2.3 Image analysis

The Xeleris GE software facilitated qualitative and semi-quantitative image analysis. We reconstructed the SPECT images with 8 mm thickness in transaxial, coronal, and sagittal slices by the Filtered Back Projection method. Moreover, we used a Chang attenuation correction and a Butterworth filter with a 0.45 cut off and order 10.

**Figure 1:**
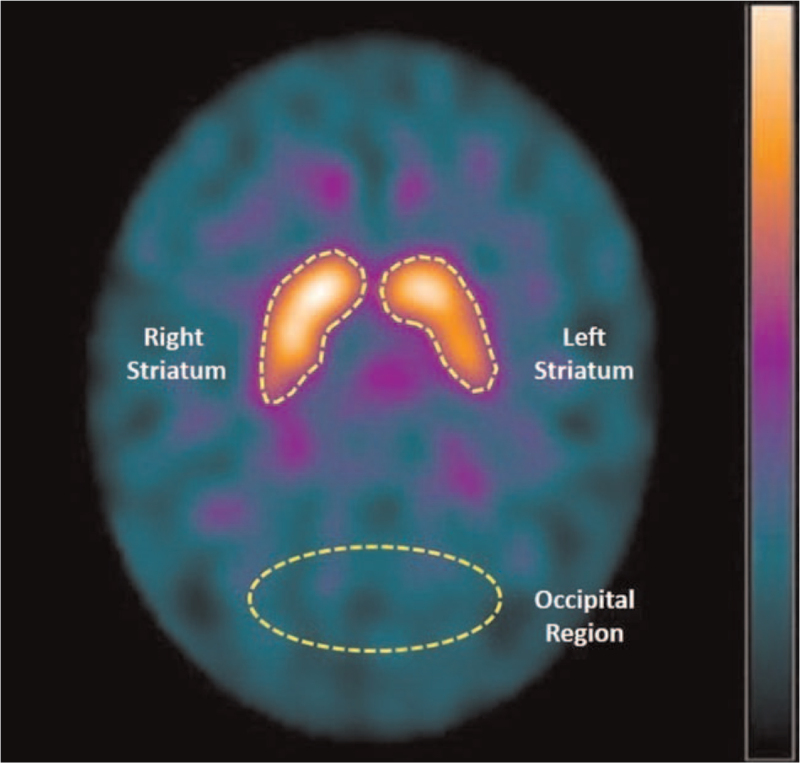
Tc-99m TRODAT-1 SPECT image of healthy volunteer, woman, 47 years old, to illustrate regions of interest drawing. Transaxial cut thickness 8 mm. Manual ROI in the right and left striatal regions and elliptical ROI in the occipital area, to calculate the DAT binding potential. Acquisition date 03/17/2007. DAT = dopamine transporter, ROI = regions of interest, SPECT = single photon emission computed tomography, Tc-99m = technetium-99 metastable.

We evaluated the images through visual inspection and quantitative evaluation of the regions of interest (ROI). We defined the striatal ROIs slightly smaller than the actual structure to avoid partial volume effects. They were drawn on 3 consecutive transaxial slices that enabled better visualization of the striatal DAT binding. We used their average to estimate the striatal concentration of DAT on the right and left sides of the brain. Thus, the ROIs were manually drawn at the striatal region with 150 to 155 pixel region with a specific binding of Tc-99m TRODAT-1 (high DAT concentration) and an elliptical drawn in the occipital lobe area with a 400 pixel region of non-specific binding of Tc-99m TRODAT-1 (low DAT concentration) (Fig. 1). We calculated the BP using the formula:

$$\text{BP}=\frac{\left[\text{STR}-\text{OCC}\right]}{\text{OCC}}$$

where, BP = binding potential; STR = striatal region, specific binding region of Tc-99m TRODAT-1 to DAT; and OCC = occipital lobe, non-specific binding region of Tc-99m TRODAT-1 to DAT.

Other modeling methods have validated the aforementioned method.^[20]^ Two investigators independently analyzed the images. They were blinded to the group conditions. However, they had been previously trained and achieved high inter-rater reliability (>0.95) with an experienced rater in the group. The rater measured the striatal DAT binding for each subject at 2 different times and achieved an intra-rater reliability of >0.95. This certified the test–retest reliability of our measurements.

### 2.4 Statistical analysis

We used the Statistical Package for the Social Sciences version 22.0 (IBM, NY) for statistical analyses. We performed a simple percentage analysis to evaluate the DAT density over age. In addition, we conducted a linear regression to determine correlations between DAT density and sex or age. A *P* value of .05 was considered statistically significant.

## 3 Results

We selected 126 images from 126 healthy subjects comprising 72 (57.1%) men and 54 (42.9%) women, aged 18 to 80 years (mean age 46.17 + 15.43 years) (Table 1). The sample showed a normal distribution (Kolmogorov-Smirnov test = 0.068; *P* = .200).

**Table 1:** Sociodemographic characteristics of Brazilian healthy individuals.

Variable	N	Mean	S.D.
Demographic	126		
Age, yrs		46.17	±15.43
Years of education		14.07	±4.41
Sex (M/F)	72/54	−	–
Dopamine transporter density			
Total striatum DAT-BP		3.25	±0.76
Left striatum DAT-BP		1.23	±0.48
Right striatum DAT-BP		1.13	±0.39

DAT-BP = dopamine transporter binding potential.

**Figure 2:**
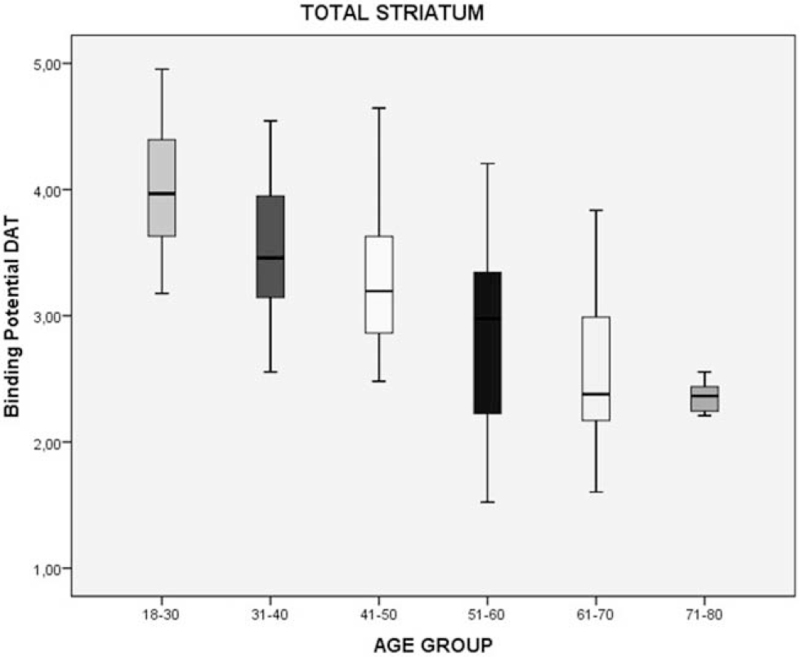
Decrease of the binding potential (BP) of the DAT in total striatum region in healthy individuals, divided by age group. DAT = dopamine transporter.

**Figure 3:**
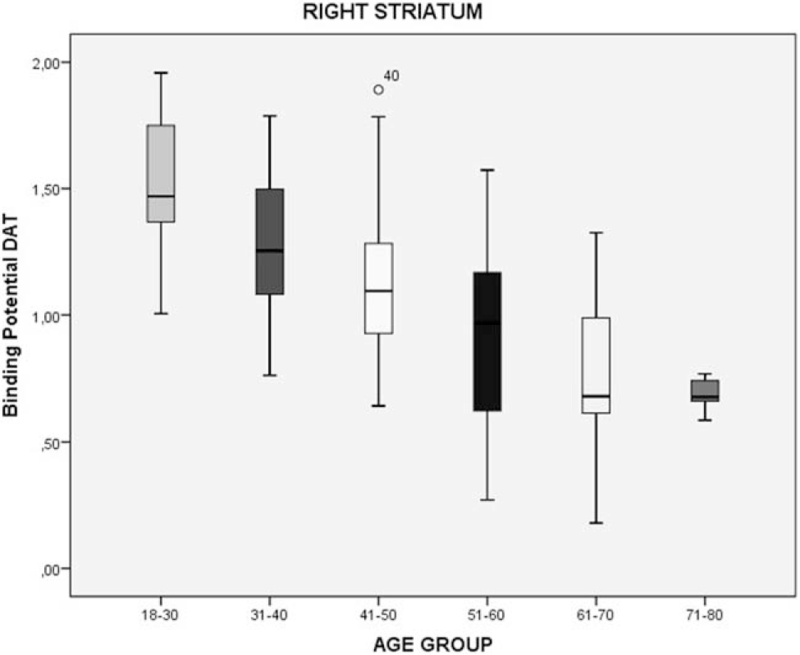
Decrease of the binding potential (BP) of the DAT in right striatum region in healthy individuals, divided by age group. DAT = dopamine transporter.

**Figure 4:**
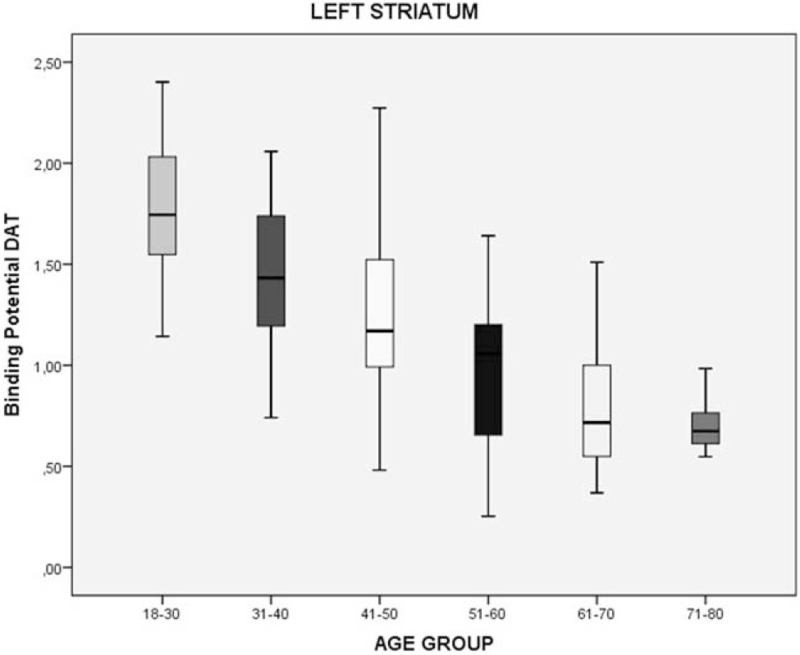
Decrease of the binding potential (BP) of the DAT in left striatum region in healthy individuals, divided by age group. DAT = dopamine transporter.

We divided the sample by age group (6 groups, separated per decade) to evaluate the DAT density by age. Considering 18 to 30 years group the standard of DAT density, we performed a simple percentage analysis. We found that the DAT density can decrease by 42%, 56%, and 60% in the total striatum, right striatum, and left striatum, respectively in the last group (71–80 years) (Table 2; Figs. 2–4).

**Table 2:** Percentage decrease of the binding potential (BP) of the DAT in striatum region in healthy individuals, divided by age group.

Age group	Regions	DAT-BP^∗^	Decrease in relation to 18–30 yrs age group
18–30 yrs	Total striatum	4.05 ± 0.46	
	Right striatum	1.54 ± 0.24	
	Left striatum	1.77 ± 0.33	
31–40 yrs	Total striatum	3.54 ± 0.58	13%
	Right striatum	1.27 ± 0.30	18%
	Left striatum	1.42 ± 0.37	20%
41–50 yrs	Total striatum	3.29 ± 0.55	19%
	Right striatum	1.15 ± 0.30	25%
	Left striatum	1.23 ± 0.37	31%
51–60 yrs	Total striatum	2.90 ± 0.66	28%
	Right striatum	0.95 ± 0.34	38%
	Left striatum	0.99 ± 0.35	44%
61–70 yrs	Total striatum	2.61 ± 0.63	36%
	Right striatum	0.80 ± 0.31	48%
	Left striatum	0.81 ± 0.33	54%
71–80 yrs	Total striatum	2.36 ± 0.13	42%
	Right striatum	0.68 ± 0.06	56%
	Left striatum	0.71 ± 0.15	60%

DAT-BP = dopamine transporter binding potential.

∗ Mean ± std. deviation.

**Figure 5:**
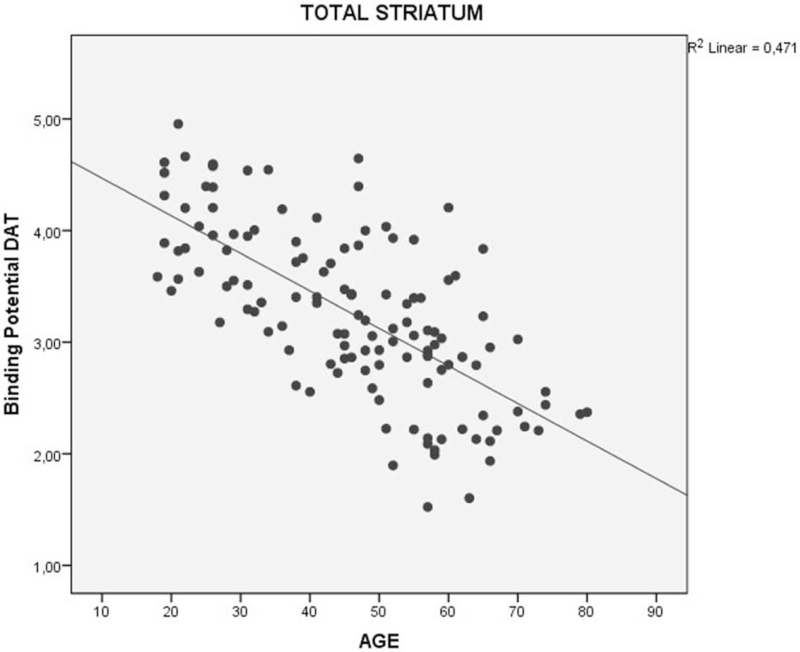
Correlation between of binding potential (BP) of the DAT and age in total striatum in healthy individuals. DAT = dopamine transporter.

**Figure 6:**
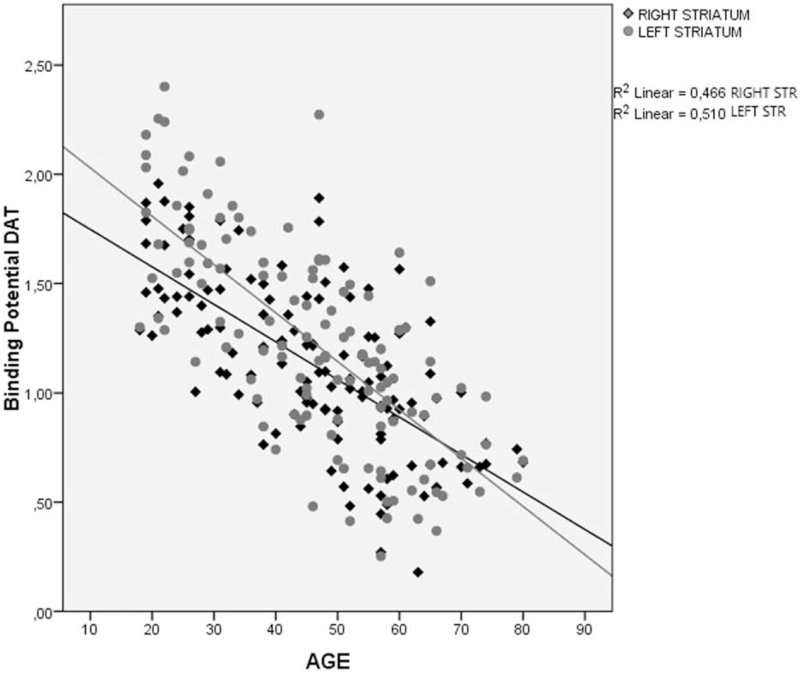
Correlation between of binding potential (BP) of the DAT and age in right striatum and left striatum regions in healthy individuals. DAT = dopamine transporter.

**Figure 7:**
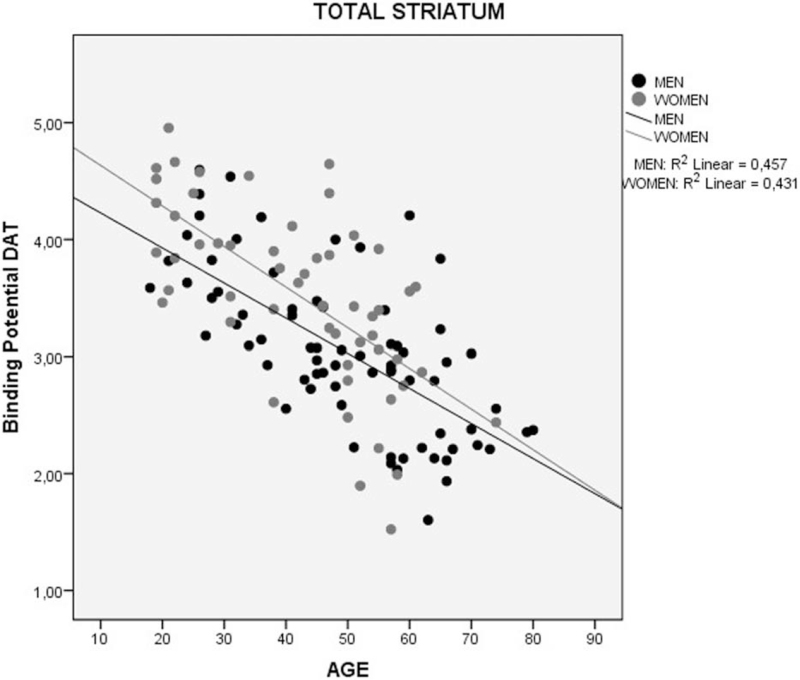
Correlation between of binding potential (BP) of the DAT and sex and age in total striatum region of healthy individuals. DAT = dopamine transporter.

Findings from the linear regression analysis revealed a significant correlation between a decrease in the binding potential of DAT (DAT-BP) and an increasing age for all regions: total striatum *R*^2^ = 0.471, *P* < .001 (Fig. 5); right striatum *R*^2^ = 0.466, *P* < .001; and left striatum *R*^2^ = 0.510, *P* < .001 (Fig. 6). Women showed a higher mean DAT-BP density than men. This was significantly correlated with a decreasing DAT and an increasing age (women *R*^2^ = 0.431 *P* < .001 vs men *R*^2^ = 0.457 *P* < .001) (Fig. 7).

## 4 Discussion

This is the first study to evaluate the DAT density in healthy Brazilians. Our results showed a decrease in the DAT density by age. Furthermore, they confirmed a persistent sex-related difference in DAT density in Brazilians.

DAT regulates the concentration of dopamine in the synaptic cleft through its reuptake into the presynaptic neurons. Moreover, it exerts an influence on dopamine function by modulating locomotor activity, cognition, and the reward system.^[21–25]^

Tc-99m TRODAT-1 selectively binds to the dopamine transporters localized at the striatum. The binding potential of the DAT (DAT-BP) corresponds to the product of the free receptor density and affinity. Furthermore, it is calculated as the ratio of striatal specific binding to the concentration of steady-state, free, and unmetabolized plasma tracer.^[26,27]^ DAT ligands, such as Tc-99m TRODAT-1 are established markers for evaluating the changes in presynaptic DAT in vivo.^[6]^

Previous studies reported on a decline in the striatal DAT at an approximate rate of 6% to 8% per decade in the human striatum,^[28–30]^ which is consistent with our results. Furthermore, we reported on a similarity in this decrease per decade in the right and left striatum. These findings corroborate the post-mortem reports of DAT loss with ageing.^[30]^ Thus, in vivo methodologies may permit the evaluation of age-related degeneration of dopamine nerve terminals, in relation to the cognitive and motor deficits that occur in normal ageing. In addition, our results also corroborate with the literature on sex-related differences in DAT density.^[31–33]^ It showed that the decrease in DAT per decade is similar in both sexes.

The use of DAT-SPECT facilitates the investigation of the presynaptic dopaminergic nigrostriatal pathway. Moreover, it is useful in clinical practice related to neurodegenerative diseases.^[34]^ PD is characterized by the selective loss of dopamine neurons in the basal ganglia and substantia nigra. However, patients with PD manifest symptoms only when 50% to 80% of the nigrostriatal neurons are lost.^[35,36]^

Clinical diagnosis sometimes fails to identify at-risk individuals before a significant loss of dopamine neurons. The reduction of DAT binding in the prodromal stage of PD suggests an early synaptic dysfunction and the activation of compensatory changes to delay the onset of symptoms.^[34,37]^ Therefore, quantitative measurements of DAT binding at baseline could predict the emergence of late-disease motor fluctuations and dyskinesias.^[37]^

Manual analysis of the images was a major limitation of our study. In addition, the images were not anatomically paired with computed tomography or magnetic resonance imaging for the ROIs. However, there were improvements in the pre- and post-processing (alignment, cut thickness, image reconstruction, attenuation corrections, and ROI delimitation). The sample includes elderly participants. Unfortunately, it wasn’t possible to perform laboratory tests to verify other clinical comorbidities. Although, most of the volunteers underwent neuropsychological assessment. We failed to obtain data regarding ethnicity and laterality of the sample, which could be helpful in future studies. Nonetheless, our sample contained 126 images of healthy individuals. Thus, a sample size strengthened our results.

## 5 Conclusion

DAT imaging is a good biomarker for evaluating the loss of dopaminergic neurons.^[38]^ Moreover, our data support the safe application of Tc-99m TRODAT-1. The sex-associated differences and age-associated changes in DAT density can explain possible dopaminergic neuromodulation. Thus, these results may facilitate our understanding of brain illnesses involving the dopamine system, such as neuropsychiatric disorders, and their associated sex-related differences. Our study revealed important findings regarding DAT-BP values in healthy Brazilians. This in turn will enable the standardization of DAT while investigating dopaminergic neurotransmission.

## Acknowledgment

The authors thank the Nuclear Medicine Department of Hospital Israelita Albert Einstein, São Paulo, Brazil, for their support in this work.

## Author contributions

All authors contributed to the study conception and design. Data collection it was made by Marilia Alves dos Reis, André C. Felício, Marcelo Queiroz Hoexter, Ilza Rosa Batista, Pedro Braga-Neto, Mariana Calzavara, Daniel Alves Cavagnolli, Cinthia Higuchi, Melissa Furlaneto Lellis Leite, Solange Amorim Nogueira, Jairo Wagner and Ming Chi Shih. Image analysis was performed by Marilia Alves dos Reis. Material preparation and statistical analysis were performed by Marilia Alves dos Reis, Ary Gadelha, Mario Luiz Vieira Castiglioni and Rodrigo Affonseca Bressan. The first draft of the manuscript was written by Marilia Alves dos Reis and all authors commented on previous versions of the manuscript. All authors read and approved the final manuscript.

**Conceptualization:** Marilia Alves dos Reis, Ary Gadelha, Rodrigo Affonseca Bressan.

**Data curation:** Marilia Alves dos Reis, Ary Gadelha, Rodrigo Affonseca Bressan.

**Formal analysis:** Marilia Alves dos Reis, Ary Gadelha, Rodrigo Affonseca Bressan.

**Funding acquisition:** Rodrigo Affonseca Bressan.

**Investigation:** Marilia Alves dos Reis, Ary Gadelha, Rodrigo Affonseca Bressan.

**Methodology:** Marilia Alves dos Reis, Ary Gadelha, André C. Felício, Marcelo Queiroz Hoexter, Ilza Rosa Batista, Pedro Braga-Neto, Mariana Calzavara, Daniel Alves Cavagnolli, Cinthia Higuchi, Melissa Furlaneto Lellis Leite, Solange Amorim Nogueira, Jairo Wagner, Mario Luiz Vieira Castiglioni, Ming Chi Shih.

**Supervision:** Ary Gadelha, Rodrigo Affonseca Bressan.

**Writing – original draft:** Marilia Alves dos Reis, Ary Gadelha, André C. Felício, Marcelo Queiroz Hoexter, Ilza Rosa Batista, Pedro Braga-Neto, Mariana Calzavara, Cinthia Higuchi, Rodrigo Affonseca Bressan.

**Writing – review & editing:** Marilia Alves dos Reis, Ary Gadelha, Rodrigo Affonseca Bressan.
